# Dataset of differentially expressed genes in mouse P12 testes in response to the loss of ATRX in Sertoli cells

**DOI:** 10.1016/j.dib.2022.108230

**Published:** 2022-05-02

**Authors:** Stefan Bagheri-Fam, Dimuthu Alankarage, Emily R. Frost, Vincent R. Harley

**Affiliations:** a Sex Development Laboratory, Hudson Institute of Medical Research, Melbourne, VIC 3168, Australia; b Department of Molecular and Translational Science, Monash University, Melbourne, VIC 3800, Australia; c Department of Anatomy and Developmental Biology, Monash University, Melbourne, VIC 3800, Australia; d Department of Biochemistry and Molecular Biology, Monash University, Melbourne, VIC 3800, Australia

**Keywords:** ATRX, Sertoli cells, Microarray, Testis development, Spermatogenesis, Androgen receptor, Imprinted genes

## Abstract

This dataset represents genes that are dysregulated in the postnatal day 12 (P12) mouse testis when ATRX is specifically inactivated in Sertoli cells (*ScAtrxKO* mice). The differentially expressed genes included in the dataset may play important roles in the testicular phenotypes observed in the *ScAtrxKO* mice, which were first reported in our previous work [1]. In fetal *ScAtrxKO* mice, Sertoli cells undergo apoptosis due to cell cycle defects, resulting in smaller testes with reduced tubule volume [1]. Adult *ScAtrxKO* mice show a wide range of spermatogenesis defects probably due to a failure of the dysfunctional ATRX protein to interact with the androgen receptor (AR) [1]. ATRX, a chromatin remodeling protein, is widely expressed in the human testis including Sertoli cells [2,3]. In XY individuals, the loss of ATRX leads to ATR-X (alpha thalassemia, mental retardation, X-linked) syndrome associated with a wide range of genital abnormalities such as hypospadias, ambiguous genitalia, and small testes with reduced tubule volume [4], [5], [6], [7], [8]. Our dataset contributes towards understanding the mechanism underlying ATRX regulation of testis development and spermatogenesis.

## Specifications Table

**Table:** 

Subject	Genetics: Developmental
Specific subject area	Molecular Biology of Sex Development
Type of data	Tables, Charts, Figures
How data were acquired	Microarray analysis using Illumina Sentrix MouseWG-6 v2.0 Expression BeadChip
Data format	Processed, analyzed
Description of data collection	The *Atrx* gene was conditionally inactivated in the Sertoli cells by crossing *Atrx^flox/flox^* mice with *AMH-Cre*/+ mice [1]. mRNA was extracted from testes at postnatal day 12 (P12) from wild-type or *Atrx* knockout (*ScAtrxKO*) male mice, prior to hybridizing to Illumina Sentrix MouseWG-6 v2.0 Expression BeadChip.
Data source location	Hudson Institute of Medical Research, Melbourne, Australia
Data accessibility	The analyzed microarray data are within this article. The raw microarray data and all supplementary files have been deposited in NCBI's Gene Expression Omnibus (GEO) [9] and are accessible through GEO Series accession number GSE195572. (https://www.ncbi.nlm.nih.gov/geo/query/acc.cgi?acc=GSE195572).

## Value of the Data

• This dataset provides a list of genes transcriptionally dysregulated in the postnatal mouse testis due to the loss of ATRX in the Sertoli cells
• The genes of this dataset are likely to play important roles within the testis and may be mutated in disorders of sex development
• Analysis of this dataset can provide valuable insights into the function of ATRX during testis development and spermatogenesis
• This dataset could be compared to postnatal microarray data from mice lacking AR specifically in Sertoli cells [10] to identify AR-dependent genes that are also regulated by ATRX
• This dataset could be compared to microarray data and quantitative RT-PCR studies from mice lacking ATRX specifically in the forebrain [11,12] to identify genes regulated by ATRX common to the testis and brain

## 1 Data Description

**Fig. 1:**
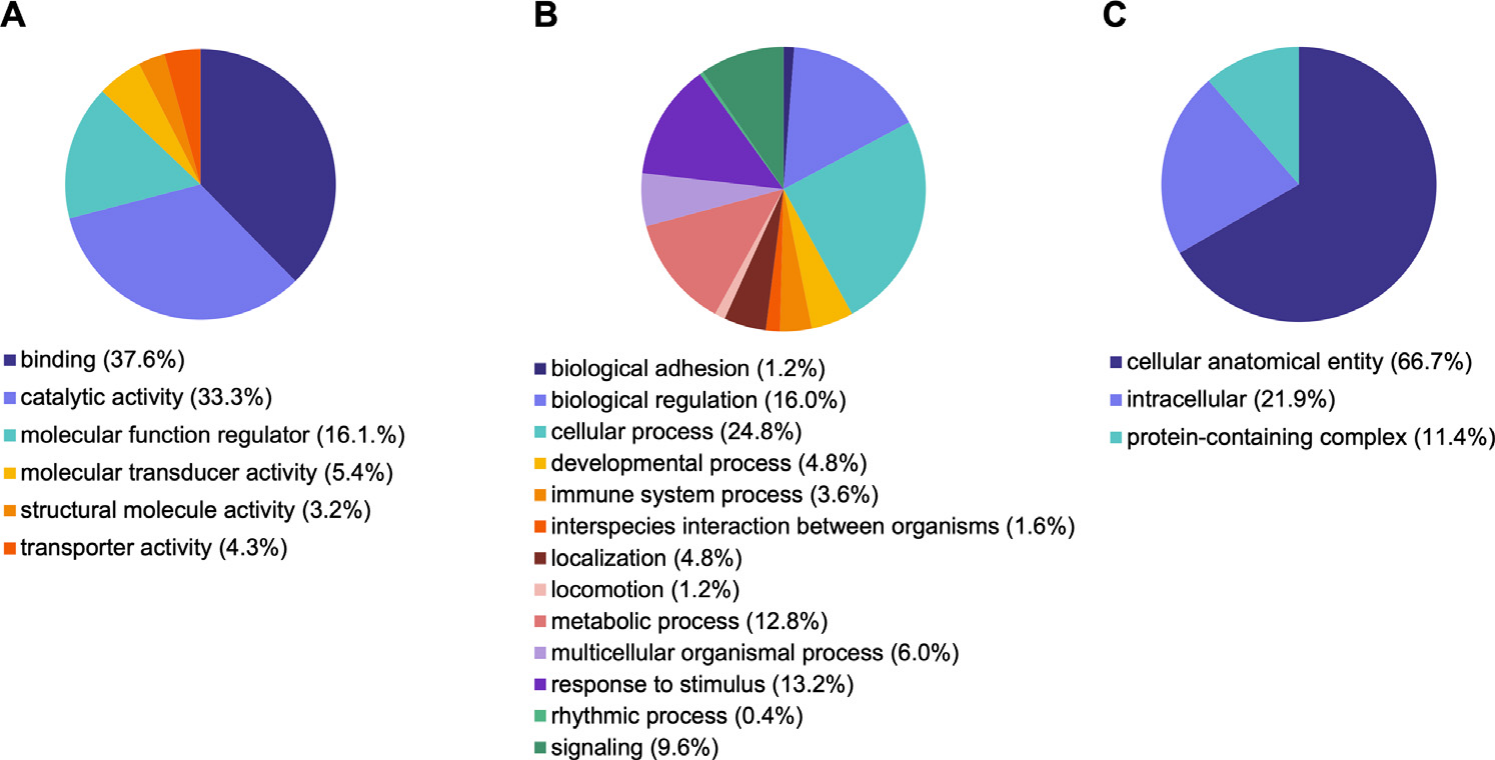
Distribution of GO terms in up-regulated genes associated with Molecular Function (A), Biological Processes (B) and Cellular Component (C).

**Fig. 2:**
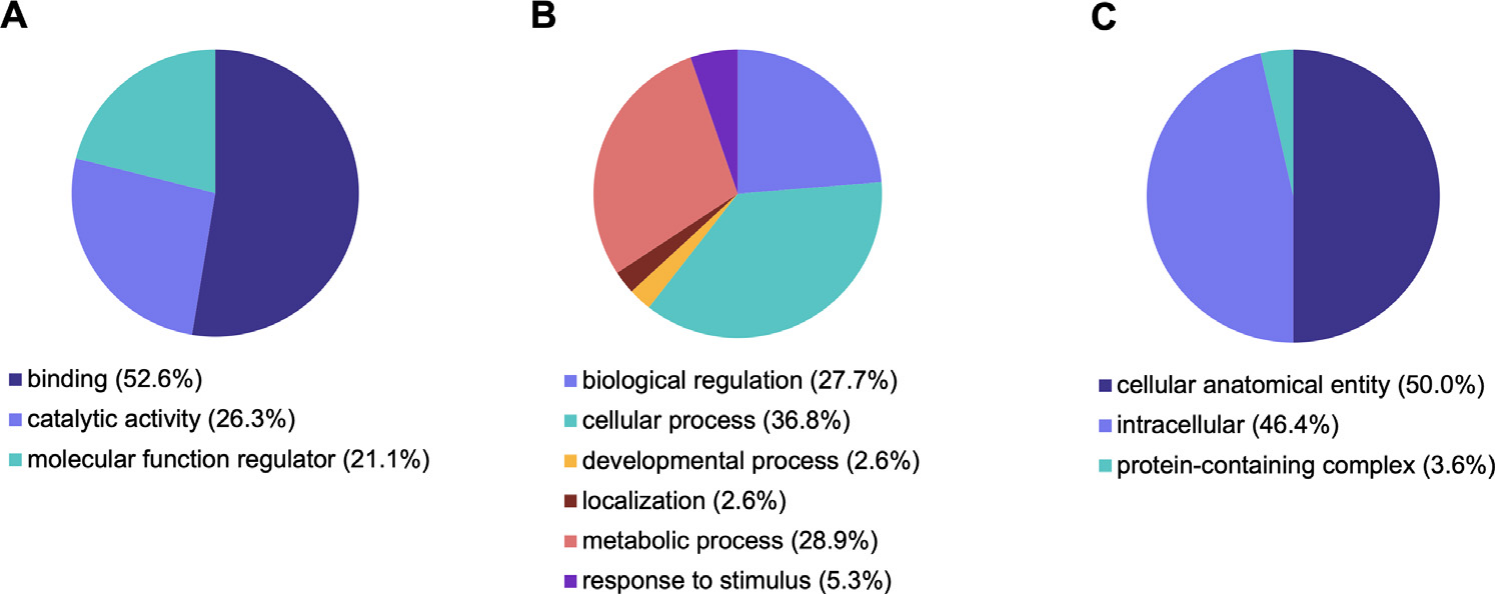
Distribution of GO terms in down-regulated genes associated with Molecular Function (A), Biological Processes (B) and Cellular Component (C).

Microarray analysis of gene expression in P12 *ScAtrxKO* testes compared to wild-type testes identified 169 differentially expressed genes (DEGs) with a ±1.5 fold expression difference that was significant (adjusted *p* < 0.05) (Supplementary Table 1; accessible through GEO Series accession number GSE195572 (https://www.ncbi.nlm.nih.gov/geo/query/acc.cgi?acc=GSE195572)). The 169 genes account for ∼0.4% of the total number of transcripts present on the Illumina BeadChip. Of the 169 DEGs, 133 transcripts were up-regulated in the *ScAtrxKO* gonads (within a range of 1.5–3.7 fold change) and 36 genes were down-regulated (within a range of −1.5–-6.4 fold change) in comparison with wild-type gonads. GO term analysis identified the most affected biological processes in up-regulated genes (Table 1) and in down-regulated genes (Table 2). The up and down regulated differentially expressed genes were annotated by association with three GO term categories: Molecular function (MF), Biological process (BP) and Cellular component (CC) (Figs. 1 and 2, Supplementary Table 2; accessible through GEO Series accession number GSE195572 (https://www.ncbi.nlm.nih.gov/geo/query/acc.cgi?acc=GSE195572)).

**Table 1:** The most affected GO terms from Biological Processes in up-regulated genes and their fold enrichment in *ScAtrxKO* testes (adjusted *p*-value ≤ 0.05).

Up-Regulated Genes			
GO Number	GO Terms	Total #	Fold Enrichment
GO:0043568	Positive regulation of insulin-like growth factor receptor signaling pathway	4	40.79
GO:0061081	Positive regulation of myeloid leukocyte cytokine production involved in immune response	4	40.79
GO:0061440	Kidney vasculature development	4	37.39
GO:0061437	Renal system vasculature development	4	37.39
GO:0030325	Adrenal gland development	5	25.49
GO:0030199	Collagen fibril organization	8	23.61
GO:0032963	Collagen metabolic process	6	18.19
GO:0044259	Multicellular organismal macromolecule metabolic process	6	17.71
GO:0035272	Exocrine system development	6	13.74
GO:0048146	Positive regulation of fibroblast proliferation	7	12.27

**Table 2:** The most affected GO terms from Biological Processes in down-regulated genes and their fold enrichment in *ScAtrxKO* testes (adjusted *p*-value ≤ 0.05).

Down-Regulated Genes			
GO Number	GO Terms	Total #	Fold Enrichment
GO:0034587	piRNA metabolic process	3	> 100
GO:0003006	Developmental process involved in reproduction	9	7.89
GO:0022414	Reproductive process	10	6.13
GO:0000003	Reproduction	10	6.12

The 133 up-regulated transcripts contain the imprinted genes *Dlk1, H19, Igf2, Cdkn1c*, and *Mest* that are silenced by ATRX in the P0 forebrain [11]. The 36 down-regulated transcripts contain *Rhox5, Spinlw1, Corin* and *Cyp2s1 -* four genes positively regulated by the AR in P10 Sertoli cells [10]. *Rhox5* and *Spinlw1* expression was validated by quantitative RT-PCR studies in P12 *ScAtrxKO* testes [1].

## 2 Experimental Design, Materials and Methods

### 2.1 Generation and Genotyping of *ScAtrxKO* Mice

Refer to [1] for method details.

### 2.2 Microarray and Statistical Analysis

500ng of each biotin labeled cRNA sample (*n* = 3 P12 *ScAtrxKO* testes, *n* = 3 P12 wild-type testes), which were produced by *in vitro* amplification of cDNA, was hybridized to Illumina Sentrix MouseWG-6 v2.0 Expression BeadChip, containing 45,281 RefSeq transcripts from the National Center for Biotechnology Information (NCBI) database (from build 36, Release 22 of the database). The Illumina microarray was performed at the Australian Genome Research Facility (AGRF) microarray service in Melbourne, Australia. The GenomeStudio software was used for initial quality control (QC), background subtraction and averaging probes to gene-level estimates, before then loading the data into R with the Bioconductor package “lumi”. This data was variance stabilized and quantile normalization was performed, then genes filtered to only those that had a detection p-value of less 0.01 in at least 1 sample; this reduced the number of genes from 30,774 down to 13,026. These genes were then tested for differential changes using the limma software package, and p-values adjusted for multiple testing using the Benjamini and Hochberg method to control the false discovery rate (FDR), then significantly changing genes selected by filtering for absolute fold change >= 1.5, and FDR<0.05. Kernel density estimates of the log2 signal intensity distributions for all six samples before and after normalization are shown in Supplementary Fig. 1, which is accessible through GEO Series accession number GSE195572 (https://www.ncbi.nlm.nih.gov/geo/query/acc.cgi?acc=GSE195572).

### 2.3 Data Annotation

Gene ontology analysis of the differentially expressed genes was performed using PANTHER Overrepresentation Test and adjusted for multiple testing by Bonferroni correction (GO Ontology database Released 2021-02-24). A total of 204 GO terms were assigned to the up-regulated genes and 4 GO terms were assigned to the down-regulated genes (adjusted *p*-value < 0.05). GO terms above *p* < 0.05 were excluded from the analysis. Differentially expressed genes that were up and down regulated were categorized by Molecular Functions, Biological Processes and Cellular Components (Figs. 1 and 2, Supplementary Table 2).

## Ethics Statements

All animal experimentation complied with the ARRIVE guidelines and the Australian code for the care and use of animals for scientific purposes (7th Edition 2004) and was approved and carried out according to the guidelines established by the Monash Medical Centre Animal Ethics Committee.

## CRediT authorship contribution statement

**Stefan Bagheri-Fam:** Conceptualization, Methodology, Validation, Investigation, Writing – review & editing, Supervision, Project administration, Funding acquisition. **Dimuthu Alankarage:** Formal analysis, Visualization, Writing – original draft. **Emily R. Frost:** Formal analysis, Visualization, Writing – review & editing. **Vincent R. Harley:** Conceptualization, Writing – review & editing, Supervision, Project administration, Funding acquisition.

## Declaration of Competing Interest

The authors declare that they have no known competing financial interests or personal relationships that could have appeared to influence the work reported in this paper.

## Data Availability

Dataset of differentially expressed genes in mouse P12 testes in response to the loss of ATRX in Sertoli cells (Original data) (GEO).

## Appendix Supplementary materials

Supplementary Table 1. Differentially expressed gene list derived from microarray of P12 *ScAtrxKO* mouse testes compared to wild-type mouse testes. Fold change cut-off is set at ±1.5 (adjusted *p* < 0.05). Image, application 1

Supplementary Table 2. GO analysis of the differentially expressed genes. Data are categorized by Biological Process (BP), Molecular Function (MF) and Cellular Compartment (CC). Image, application 2
